# Dopamine transients follow a striatal gradient of reward time horizons

**DOI:** 10.1038/s41593-023-01566-3

**Published:** 2024-02-06

**Authors:** Ali Mohebi, Wei Wei, Lilian Pelattini, Kyoungjun Kim, Joshua D. Berke

**Affiliations:** 1 Department of Neurology, University of California San Francisco, San Francisco, CA USA; 2 Department of Psychiatry and Behavioral Sciences, University of California San Francisco, San Francisco, CA USA; 3 Neuroscience Graduate Program, University of California San Francisco, San Francisco, CA USA; 4 Kavli Institute for Fundamental Neuroscience, University of California San Francisco, San Francisco, CA USA; 5 Weill Institute for Neurosciences, University of California San Francisco, San Francisco, CA USA

**Keywords:** Learning and memory, Psychology, Reward, Neurotransmitters

## Abstract

Animals make predictions to guide their behavior and update those predictions through experience. Transient increases in dopamine (DA) are thought to be critical signals for updating predictions. However, it is unclear how this mechanism handles a wide range of behavioral timescales—from seconds or less (for example, if singing a song) to potentially hours or more (for example, if hunting for food). Here we report that DA transients in distinct rat striatal subregions convey prediction errors based on distinct time horizons. DA dynamics systematically accelerated from ventral to dorsomedial to dorsolateral striatum, in the tempo of spontaneous fluctuations, the temporal integration of prior rewards and the discounting of future rewards. This spectrum of timescales for evaluative computations can help achieve efficient learning and adaptive motivation for a broad range of behaviors.

## Abstract (web summary)

Mohebi et al. report that dopamine (DA) pulses in different rat striatal subregions signal prediction errors across different timescales. In this way, one learning process may achieve a range of adaptive behaviors.

## Main

Animal behavior is frequently driven by expectations of future rewards. The nature of these expectations, and how they are updated, is a central question in behavioral neuroscience. One important source of information about future rewards is past rewards. For example, if a course of action has been producing rewards at a high rate, it may be worth continuing, rather than allocating time to alternatives^1^. Reward rate can be tracked as rewards received over some window of recent history^2,3^.

Animals also learn that certain cues and contexts are predictive of reward. In reinforcement learning (RL) theory^4^, agents make a prediction of reward (‘value’) for each situation (‘state’) they encounter. As they experience events that are better or worse than expected, they generate a reward prediction error (RPE) that is used to update the values associated with prior states. RL algorithms have been highly influential because they can produce effective artificial learning systems and because RPE signals appear to be encoded by brief fluctuations in the firing of midbrain dopamine (DA) cells^5–7^. DA cells project widely but especially to the striatum, a key brain node for value-guided decision-making^8,9^. RPE-scaled striatal DA release^10,11^ may engage synaptic plasticity^12,13^ to update values and thereby influence subsequent behavior.

Predicting rewards involves specifying a timescale. In many models, this timescale is set by a discount factor—how rapidly rewards decline in value further in the future. It makes sense to discount rewards that are far away in time—because they are less certain to occur at all and because working for a distant reward can mean foregoing more immediate opportunities^14^. Yet some rewards are worth taking considerable time and effort to acquire. To maintain motivation and avoid choosing less favorable, but faster, gratification, delayed rewards must not be discounted too quickly. Excessive discounting—that is, failure to maintain a sufficiently long *time horizon* when making decisions—has been reported in a range of human psychiatric disorders^15^, notably drug addiction^16^.

DA RPEs have been classically considered a uniform, widely broadcast scalar signal^5,17^. A single RPE signal implies a single underlying value, based on a single discount rate, and so defines a single timescale for learning and decision-making. By contrast, animals need to make decisions, assess outcomes and update their behavior accordingly over multiple timescales. During rapid production of motor sequences (for example, birdsong), desirable results are produced by patterns of muscle activation a small fraction of a second before^18^; it would be maladaptive to assign credit to actions performed much earlier. By contrast, other behaviors such as hunting can take orders of magnitude longer to complete and receive feedback^14^. Evaluation using multiple timescales in parallel can better account for animal behavior^19–21^ and also improve the performance of artificial learning systems^22,23^.

Furthermore, there is now substantial evidence for heterogeneity of DA cell firing^24,25^ and DA release across distinct striatal subregions^11,26–29^. These subregions are components of distinct large-scale loop circuits^30^, proposed to serve as distinct levels of a hierarchical RL architecture^31^. Specifically, more dorsal/lateral striatal subregions are concerned with briefer motoric details, whereas more ventral/medial areas help to organize behavior over longer timescales^32^. Theoretical studies have proposed a corresponding gradient of temporal discount factors across the striatum^19^. However, the existing evidence for graded discounting is sparse and inconsistent^33–35^.

Here we report multiple lines of evidence for a gradient across the striatum of the timescales that determine DA dynamics. We focus especially on transient (phasic) DA responses to reward-predictive cues, which we show differ substantially between subregions. We demonstrate that these differences can be largely explained by underlying predictions that use different timescales to track prior rewards and discount future rewards. This portfolio of time horizons may enable animals to make a variety of adaptive decisions within complex environments.

## Results

### DA tempo depends on striatal subregion

**Fig. 1:**
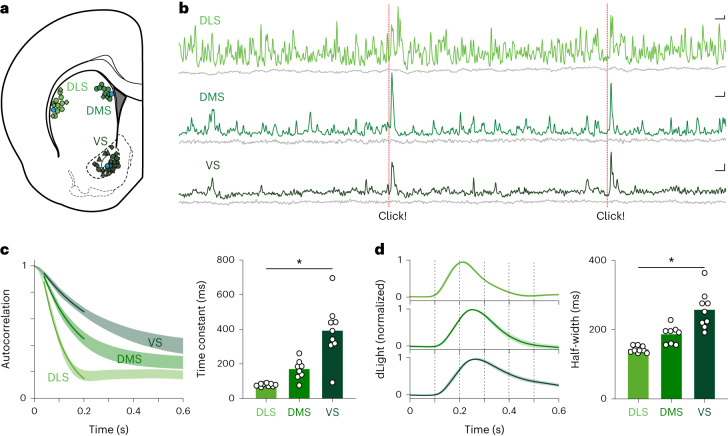
DA tempo depends on striatal subregion. **a**, Rat brain atlas section^86^ showing approximate locations of fiber optic tips (circles) within striatal subregions. Blue circles indicate the locations for the recordings in **b**. Symbols indicate recording locations for behavioral tasks as follows: circles indicate both instrumental and Pavlovian tasks^11^, triangles indicate instrumental only and diamonds indicate the multiple delay task. For further details, see Extended Data Fig. 1. **b**, Example showing simultaneous, raw dLight photometry from each subregion in an awake unrestrained rat, outside of specific task performance. Green traces indicate DA signals (470 nm), and gray traces indicate corresponding control signals (interleaved 415 nm measurements). Occasional randomly timed sugar pellet deliveries are marked as ‘Click!’ (the familiar food hopper activation sound). Scale bars: 1 s, 1% Δ*F*/*F*. **c**, Left, average autocorrelogram functions for spontaneous dLight signals in each subregion. Bands show mean ± s.e.m., and darker lines indicate best-fit exponential decay for the range of 40–200 ms. Data are from *n* = 13 rats over 15 recording sessions each; fiber placements *n* = 9 DLS, *n* = 8 DMS, *n* = 9 VS. Right, decay time constant depends on subregion (one-way ANOVA: *F*(2, 23) = 22.9, *P = 3.4 × 10^−6^). **d**, Left, average dLight signal change after an unexpected reward click. Right, duration (at half maximum) of signal increase depends on subregion (one-way ANOVA: *F*(2, 23) = 24.2, *P = 2.2 × 10^−6^). To facilitate comparison between rats and regions, the dLight signal is normalized to the mean peak response (within 1 s) to uncued reward delivery. ANOVA, analysis of variance.

**Extended Data Fig. 1:**
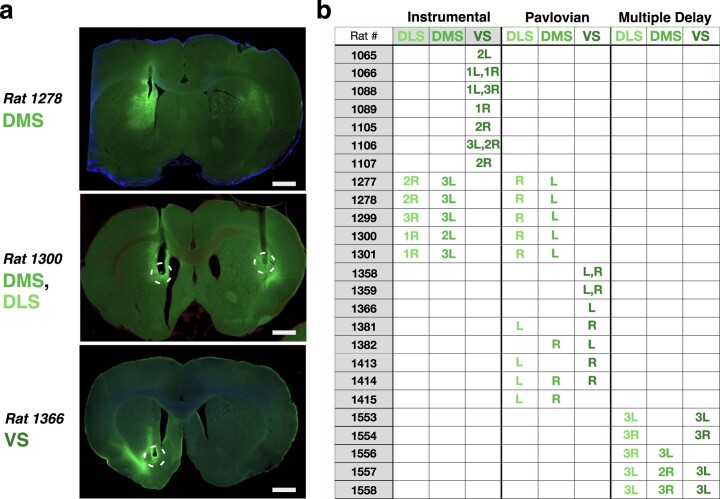
Photometry recording locations. **a**, Histology examples showing optic fiber tip locations (circled) and dLight1.3b expression (green), in DLS, DMS, VS. Scale bar: 1 mm. For all fiber placements, see Fig. 1a. **b**, Table showing included fiber subregions for each rat and task. “L” indicates left hemisphere, and “R” indicates right hemisphere. For the instrumental task, numbers (1–3) indicate that multiple sessions were included for that fiber placement. A subset of data from rats 1065–1107 were previously reported^11^.

We used fiber photometry of the fluorescent DA sensor dLight1.3b^11^ to observe DA release fluctuations in the striatum of awake, unrestrained rats. We tested the following three standard subregions (Fig. 1a and Extended Data Fig. 1): dorsolateral (DLS), dorsomedial (DMS) and ventral (VS; targeting the core of the nucleus accumbens). These receive distinct patterns of cortical input^36^ and are often considered to have distinct ‘motor,’ ‘cognitive’ and ‘limbic’ functions, respectively^37,38^.

We first examined spontaneous DA fluctuations, unconstrained by task performance. DA dynamics were clearly different in each subregion (Fig. 1b and Supplementary Video 1). DLS signals showed near-constant, rapid change, whereas VS signals evolved more sporadically and slowly^39,40^ (Fig. 1c). When presented with a familiar, but unexpected, reward cue—the click of a hopper dispensing a sugar pellet—all three subregions showed a DA transient. This transient was briefest in DLS and lasted longest in VS (Fig. 1d). Previous voltammetry studies reported that this same reward cue evoked DA selectively in VS^26^, but our use of dLight may have revealed DLS/DMS responses that are too brief to readily detect with voltammetry. Briefer DA signals in more dorsal regions are consistent with studies showing faster rates of DA uptake, across species^41,42^, although this alone appears insufficient to explain the highly distinct spontaneous DA events in simultaneous recordings (Fig. 1b).

### Distinct timescales for tracking reward history

**Fig. 2:**
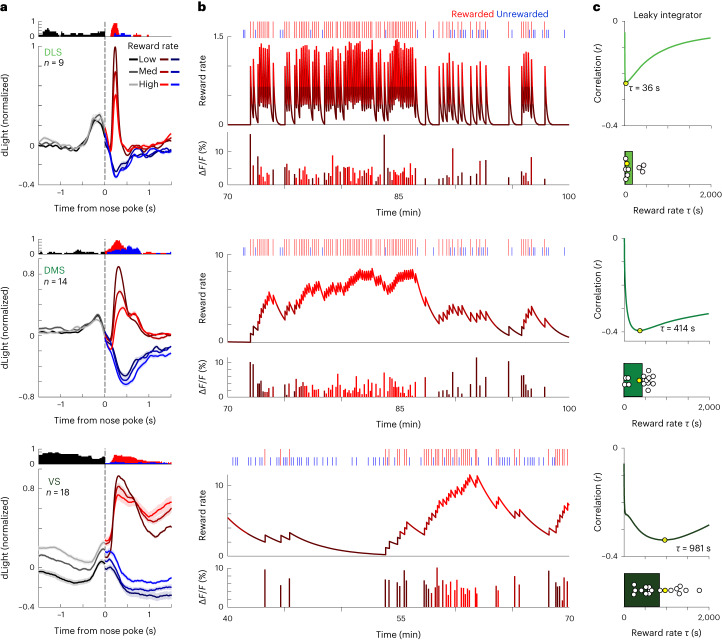
DA prediction errors depend upon subregion-specific reward history timescales. **a**, Mean dLight DA signals aligned on the instrumental task nose poke that may trigger reward delivery click (red) or not (blue). Signals are normalized to the peak DA response within 1 s of unpredictable reward deliveries (later in the same recording session) and broken down by recent reward rate (in terciles), with higher reward rates in brighter colors. Histogram above each plot shows the fraction of signals that significantly depended on reward rate (linear regression, *P* < 0.01), consistent with RPE coding after nose poke. Data are from 12 rats, 1–3 sessions each (see Extended Data Fig. 1 for targets in each rat). Reward rates were calculated using a leaky integrator of reward receipts (Methods), choosing the *τ* parameter for each subregion separately to maximize RPE coding (alternative models of reward prediction or behavioral fits gave similar results; Extended Data Fig. 2). The bump before nose poke (most prominent for DLS) is the DA response to an earlier Go! cue, smeared by variability in reaction and movement times. **b**, Portions of example recording sessions, for each subregion. Top, sequence of trial outcomes (rewarded trials indicated by tall red ticks, unrewarded by short blue ticks). Middle, corresponding reward rate estimated with a leaky-integrator model. Graphs are color-coded by the terciles of the reward rate. The decay parameter *τ* was chosen to maximize the (negative) correlation between the reward rate and the DA response to the reward clicks (bottom, peak DA change within 1 s of reward click). **c**, For each subregion: the top panel shows the correlation between DA values and reward rate as a function of the decay parameter *τ*, for the corresponding reward rate plot in **b**; the bottom panel shows best-fit *τ* for all individual sessions. The best-fit decay parameter varies by subregion (repeated measures ANOVA, *F*(2, 39) = 23.6, *P* = 2.0 × 10^−5^). The strongest correlations are seen in DLS with a shorter time horizon (small *τ*) and in VS with a longer time horizon (large *τ*).

**Extended Data Fig. 2:**
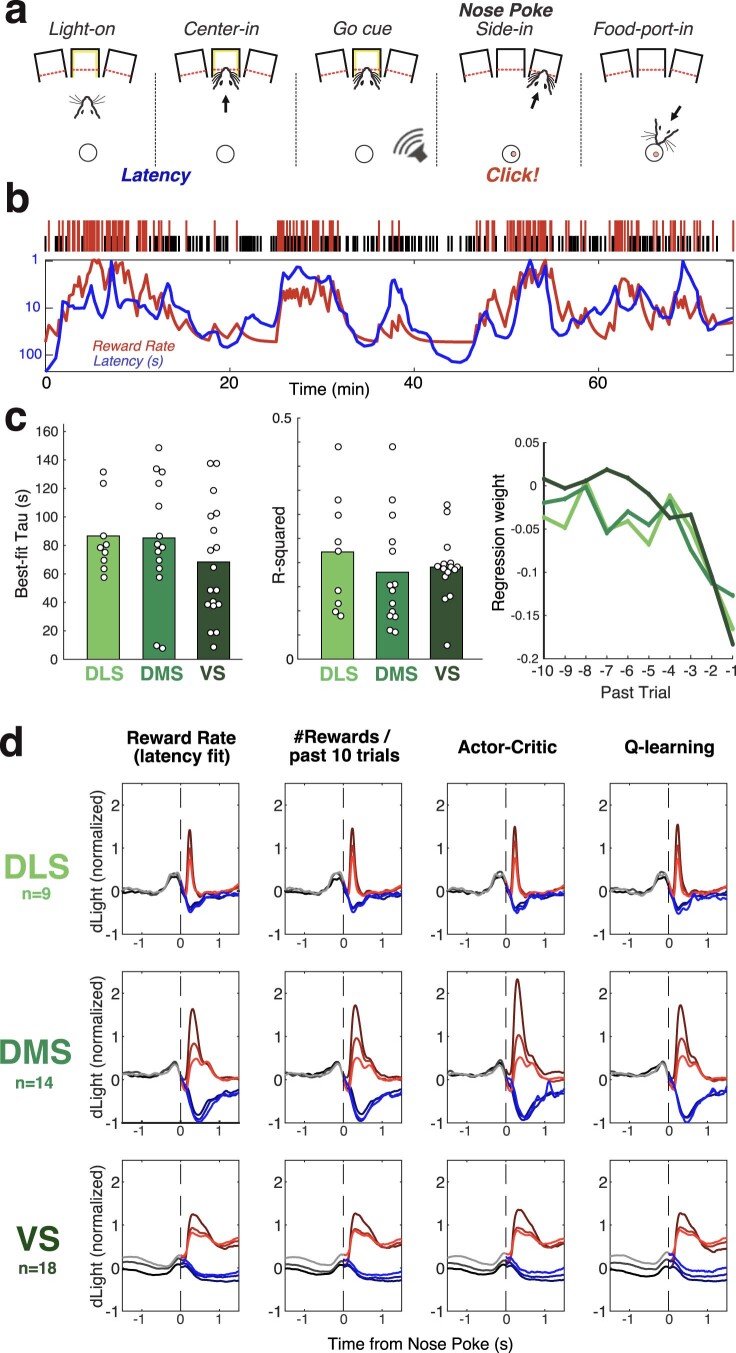
Instrumental behavior and alternative RPE fits. **a**, Schematic of instrumental task events. Here we focus on DA signals following the nose poke at Side-In, when the rat discovers if the current trial will be rewarded (food hopper click) or not (for information about other events, see refs. ^11,43^). As a measure of reward expectation, we use “latency” (the time between initial light on and the rat’s center-in nose poke. **b**, Example behavioral session showing fit between latency (log scale, inverted) and recent reward rate. Tick marks at top show the timing and outcome of each trial (taller red ticks indicate rewarded trials, shorter black ticks unrewarded). Graphs show latency (5-trial running average) and reward rate, calculated with a leaky integrator using the τ parameter that produced the strongest (negative) correlation between latency and reward rate. **c**, Left, best-fit τ (to maximize the absolute correlation between reward rate and latency) for each session in which DLS, DMS and/or VS signals were recorded. There was no significant behavioral difference between recording locations (repeated measures ANOVA, F(2,39) = 1.72, p = 0.197). Middle, the amount of variance in latency that was explained by best-fit reward rate did not differ by recording location (repeated measures ANOVA, F(2,39) = 0.180, p = 0.673). Right, Coefficients of multiple regression examining effects of the outcome of the preceding 10 trials on (log) latency, separately for each subregion (same colors as bar charts). **d**, Alternative estimates of reward expectation produce similar RPE results. Each column uses the same data and format as Fig. 2a. From left, “reward rate” is also based on a leaky integrator, but using the τ best-fit to latency (as in B/C). “#Rewards in the past 10 trials” is a simple count. “Actor-critic” uses the Critic value from a trial-based actor-critic model, fitting the critic learning rate to behavioral latency and the actor α, β parameters to left and right choices. Q-learning uses a trial-based Q-value model, fitting the α and β parameters to choices and using Q (chosen action) as reward expectation.

As DA transients can signal RPE, we next examined how the response to this reward click in each area is affected by changing reward expectation. We took advantage of an instrumental task that we have described extensively in previous work^11,43^. Well-trained rats make nose pokes, which sometimes produce the reward delivery click; reward probabilities shift without warning between 10% and 90% (see Extended Data Fig. 2 for task details). Rats adapt their behavior accordingly; in particular, they are more motivated to initiate trials when the recent reward rate is high (Extended Data Fig. 2b,c). As previously reported, this higher reward expectation also reduces the VS DA response to reward delivery (Fig. 2a, bottom), consistent with (positive) RPE coding. We observed this pattern in DLS and DMS too (Fig. 2a, top and middle), although the DA transient was briefer in DMS compared to VS and again remarkably brief in DLS (mean half-width 121 ± 16 ms s.e.m.).

Expectations of future rewards can reflect past reward history over a range of possible timescales^44^. Although all subregions showed a DA transient to the reward cue, this was not a ‘global’ RPE signal—it did not reflect the same underlying reward history timescale in each subregion. To reveal this, we first estimated the reward rate using a simple ‘leaky integrator’ of rewards^2^. This model has a single parameter *τ*—larger *τ* corresponds to a longer timescale, allowing rewards to better summate over multiple trials (Fig. 2b). For each recording site, we determined the *τ* that produced the strongest correlation between DA transients and RPE (Fig. 2c, upper plots). We observed a systematic relationship to location—best-fit *τ* was shortest in DLS, intermediate in DMS and longest in VS (Fig. 2c, lower plots), consistent with a spectrum of timescales for reward rate estimation. This relationship to location was observed despite similar behavioral measures of reward expectation in the corresponding recording sessions (Extended Data Fig. 2).

**Extended Data Fig. 3:**
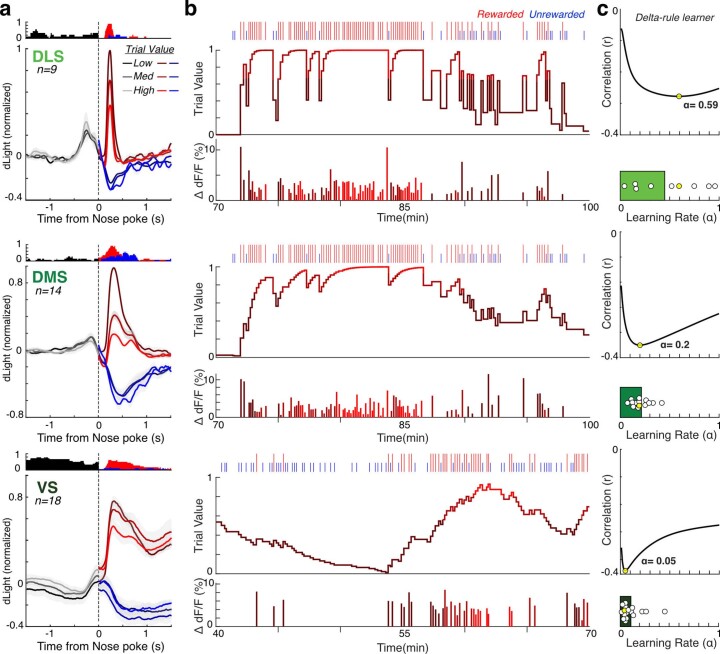
Comparing reward rate timescales using a delta-rule learner. **a,b,c** Identical to Fig. 2a,b,c but using a delta-rule to track reward rates, instead of a leaky integrator. This model updates once per trial, rather than continuously in time. The learning rate *α* that maximizes correlation between RPE and DA at reward delivery significantly varies by subregion (one-way ANOVA, F (2, 39) = 23.2, p = 2.2 x 10^-5^). The strongest correlations are seen in DLS with a larger learning rate (that is faster forgetting of trial history) and in VS with a smaller learning rate (that is tracking a more extended history of outcomes).

As an alternative measure of the extent of recent history used to estimate upcoming reward, we considered how much reward estimates are updated by the outcome of each trial^24,45^. Smaller updates (lower ‘learning rate’) produce dependence on outcomes over a longer history of trials^46^. We determined the learning rate *α* that maximized DA—RPE correlations at the reward click (Extended Data Fig. 3). Best-fit *α* was highest in DLS and lowest in VS (Extended Data Fig. 3c), again indicating that VS is concerned with reward rates estimated over more prolonged timescales.

### Region-specific responses to reward-predictive cues

**Fig. 3:**
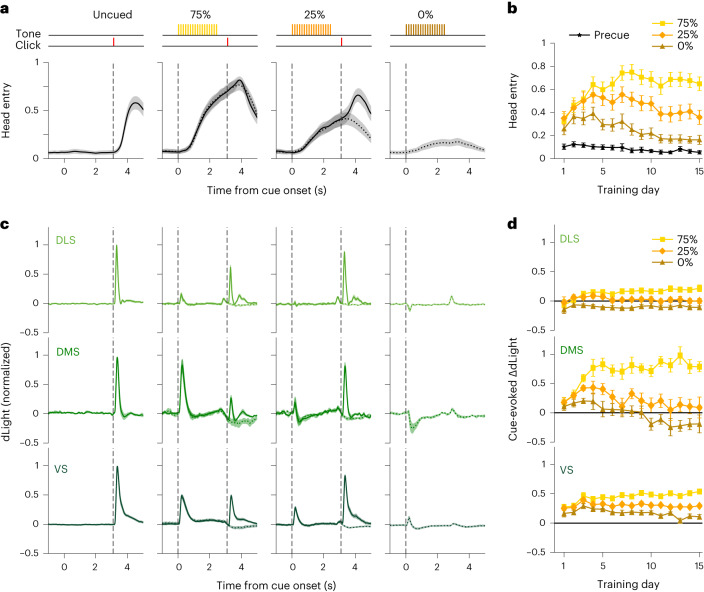
Subregion-specific DA responses to reward-predictive cues. **a**, Top, the Pavlovian task consists of four trial types, selected at random, with differing reward probabilities. Bottom, after training, cues increase anticipatory head entries into the reward port (fraction of trials with beam breaks at each instant, mean ± s.e.m.), and this scales with reward probability. Data shown are averages from training days 13–15, for *n* = 13 rats. **b**, During early training days, rats increase their behavioral responses to all cues, before progressively learning to discriminate between cues (error bars, s.e.m.; two-way repeated measures ANOVA showed a significant CUE × DAY interaction, *F*(28, 336) = 12.3, *P* = 0.0001). Points show average head entry in over a 0.5 s epoch just before cue onset (black) or just after cue offset (colors; that is immediately before the time that reward could be delivered). **c**, Average dLight signal change for each trial type after training (days 13–15; *n* = 13 rats with fibers in DLS (*n* = 9), DMS (*n* = 8) and VS (*n* = 9)). Solid lines indicate rewarded trials, and dotted lines indicate unrewarded. **d**, Time course of DA increases to each cue in each subregion over training (mean ± s.e.m.). By the late stage of training (days 13–15), the mean DA response depended on both cue identity and subregion (two-way ANOVA, significant CUE × AREA interaction, *F*(4, 66) = 6.4, *P* = 0.0002). For more details on the development of behavior and DA responses, see Extended Data Fig. 4.

**Extended Data Fig. 4:**
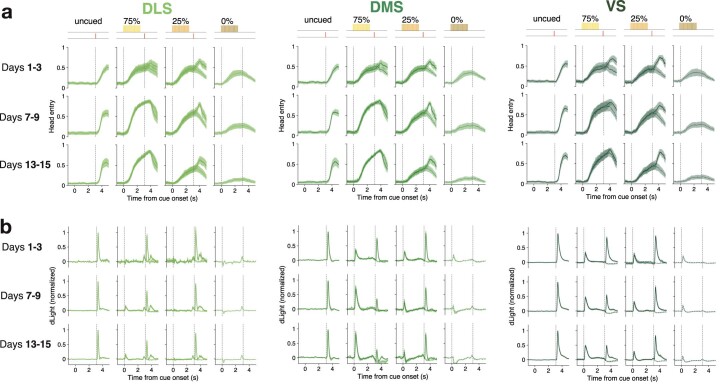
Development of approach behavior and DA cue responses in each subregion. **a**, Head-entry behavior develops in a very similar way regardless of recording site. Data shown are averaged across days 1–3, 7–9 or 13–15, respectively. **b**, Same sessions as **a**, but showing mean DA responses during each trial type. In all subregions, discrimination between cues increases with time, but this is slow in VS. Data are presented as mean ± SEM.

Beyond simply tracking past reward rate, animals can also learn that specific cues are predictive of future rewards. The RPE theory of DA function was developed based largely on DA cell responses to Pavlovian conditioned cues that predict individual future rewards^5,7^. We therefore examined DA cue responses during acquisition and performance of a Pavlovian approach task (Fig. 3a). Auditory cues (trains of 2, 5 or 9 kHz tone pips) predicted the reward delivery click a few seconds later, with distinct probabilities (75%, 25% and 0%; Methods). Each trial presented one of the cues, or an uncued reward delivery, in random order, with a 15–30 s delay between trials. Rats were trained for 15 d, with 60 trials of each type per day. Early on, all cues increased the likelihood that rats would approach and enter the food hopper (Fig. 3b), consistent with generalization between cues^47^. Over the course of training (3,600 trials total), rats showed increasing discrimination, entering the food hopper in proportion to cued reward probability (Fig. 3b and Extended Data Fig. 4).

These Pavlovian cues evoked strikingly different DA responses in each subregion (Fig. 3c,d). By the end of training, DMS DA showed strong RPE coding—the 75% cue produced a strong DA transient, the 25% cue a much smaller increase and the 0% cue a transient dip in DA (Fig. 3c). VS DA cue responses also scaled with RPE, but showed worse discrimination between cues, particularly on early training days, and remained positive for all cues throughout the 15 d of training (Fig. 3d and Extended Data Fig. 4). Concordant results of VS DA increases to a learned 0% cue (CS−) have been previously observed and attributed to generalization between cues^48^. Finally, in DLS all cues evoked much smaller DA responses (relative to unpredicted reward delivery). This did not simply reflect a failure of DLS-related circuits to learn—the DLS DA transient at reward delivery was substantially diminished if preceded by the 75% cue (Fig. 3c), consistent with an acquired reward prediction.

### Weak DLS cue responses reflect very fast discounting

**Fig. 4:**
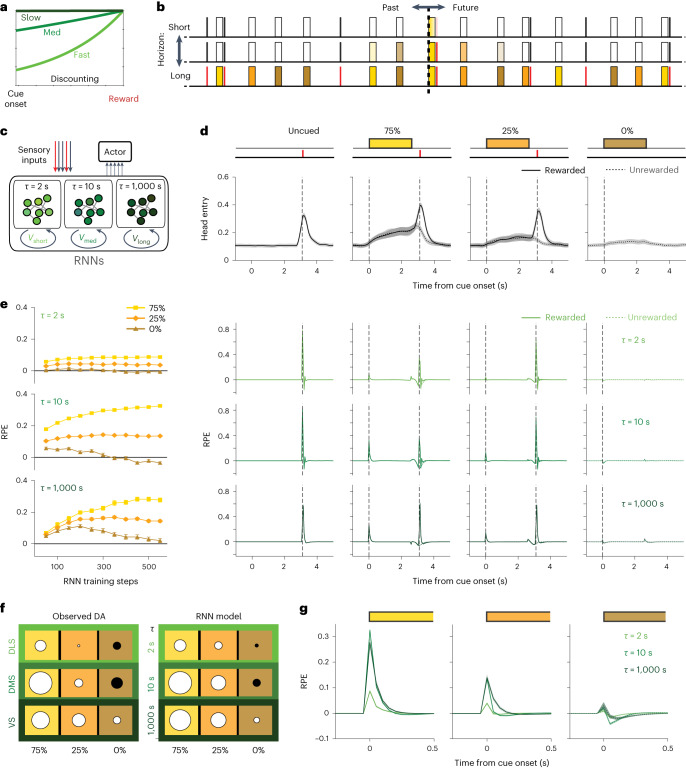
A longer time horizon accounts for slower VS cue discrimination. **a**, Faster temporal discounting erodes the value indicated by the onset of a reward-predictive cue, even if the reward is certain to appear. **b**, Schematic representation of part of a long random sequence of trials within a single training session, with colors indicating the cue in each trial. At any given moment, an RL agent may be estimating the amount of reward that is coming ‘soon’ and updating such estimates based on what happened ‘recently.’ If the time horizon is long, ‘soon’ can encompass expected rewards across multiple trials, even if the current trial has a 0% chance of reward. **c**, Schematic representation of RNN model, with three distinct pools of LSTM units. Each pool receives the same sensory inputs but maintains its own value output based on a distinct timescale ($\tau$ = 2 s, 10 s or 1,000 s; $\tau$ is related to discount factor *γ* by $\gamma ={e}^{-{{{{\rm{d}}}t}}/\tau }$, where d*t* is the time step size). All three pools project to the Actor, which generates the probability of nose-poking. **d**, Model poke probability (top) and temporal-difference RPEs for each LSTM pool, after 550 training steps. Data are presented as mean ± s.e.m., average over 20 simulations with different seeds. **e**, Development of RPEs at cue onsets across training (mean ± s.e.m., average over 20 simulations; see Extended Data Fig. 6 for extended training). **f**, Comparison of the pattern of relative sizes of responses, using RPEs from the RNN model (after 550 training steps) and observed rat DA responses (averaged across days 13–15). For both model and dLight data, the largest circle size corresponds to the largest response. Circle area is proportional to the cue response amplitude with white for positive and black for negative responses. **g**, Close-up view of the cue responses shown in **e**.

**Extended Data Fig. 5:**
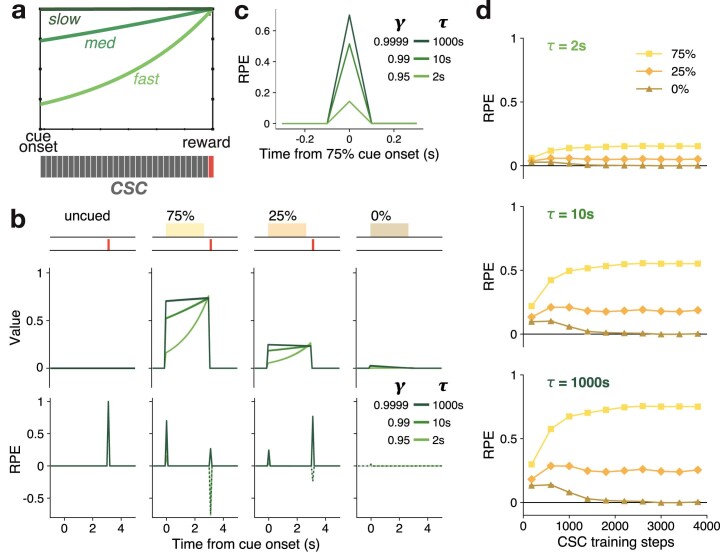
Faster temporal discounting can explain weaker DLS cue responses. **a**, In the CSC model, the cue-reward interval is divided into a fixed set of brief sub-states (we used 100 ms duration). **b**, Values and corresponding temporal-difference RPEs for the CSC model after training in the Pavlovian task (step 3800). Discount factor γ was set to 0.95 (light green, “fast”), 0.99 (mid-green) or 0.9999 (dark green, “slow”). With a time step of 100 ms, these correspond to an exponential time constant (*τ*) of 2 s, 10 s and 1000 s, respectively (*γ* = exp(−dt/τ)). Even if the cued reward probability is high (75%), RPEs at cue onset are weaker when the discount factor is lower (RPEs at reward delivery are unchanged). **c**, Close-up of the CSC RPE response to the 75% cue. **d**, Development of RPEs at cue onsets with training. Note that cue discrimination is larger if γ is closer to 1 (plotted in more detail in Extended Data Fig. 6). Overlapping cue representations cause this CSC model to produce a positive RPE to the 0% cue early in training, but this fades to zero with extended training.

We reasoned that these distinct subregional patterns could also reflect distinct time horizons for value computations. If future rewards are discounted especially fast in DLS-related circuits, even a brief delay would substantially diminish the value indicated by cues (Fig. 4a). To assess this potential explanation for our results, we turned to computational models that address the evolution of value within trials. We first applied a standard, simple model in which the cue-reward interval is divided into a regular sequence of sub-states (the complete serial compound (CSC)^49^; Extended Data Fig. 5a). Over the course of learning, value propagates backward along the sub-state chain^50^. As expected, when we compared model versions with distinct discount rates, rapid discounting reproduced the DLS pattern of smaller cue responses despite a cue-dependent response to reward delivery (Extended Data Fig. 5b–d). Including overlap between cue representations allowed the CSC to also reproduce generalization between cues early in training (Extended Data Fig. 5d).

However, this CSC model of the cue-reward interval could not readily account for the slower, poorer cue discrimination in VS (Extended Data Fig. 5d) and is incapable of reproducing the negative response to the 0% cue we saw in DMS. This model is not designed to handle prolonged time horizons that might span multiple trials^51^ (Fig. 4b). Furthermore, the splitting of experience into discrete, equally fine sub-states becomes ever more artificial as intertrial intervals get longer and more variable^52,53^.

### Slow discounting impedes cue discrimination by VS DA

We therefore turned to an alternative approach for estimating the evolution of values, using recurrent neural networks (RNNs)^54,55^. In our composite RNN model (Fig. 4c; Methods), three subnetworks use RL to generate distinct values in tandem^56^, each based upon a distinct discount rate. The model has no discrete states and time is not explicitly represented, but rather is implicit within network population dynamics^57^. With the sole assumption that discounting is fastest in ‘DLS’ and slowest in ‘VS,’ the RPEs generated by the model recapitulated key distinct features of striatal DA transients (Fig. 4e–g). These include the diminutive DLS responses as before, but also the negative DMS response to the 0% cue, and poor VS cue discrimination compared to DMS (especially earlier in training).

**Extended Data Fig. 6:**
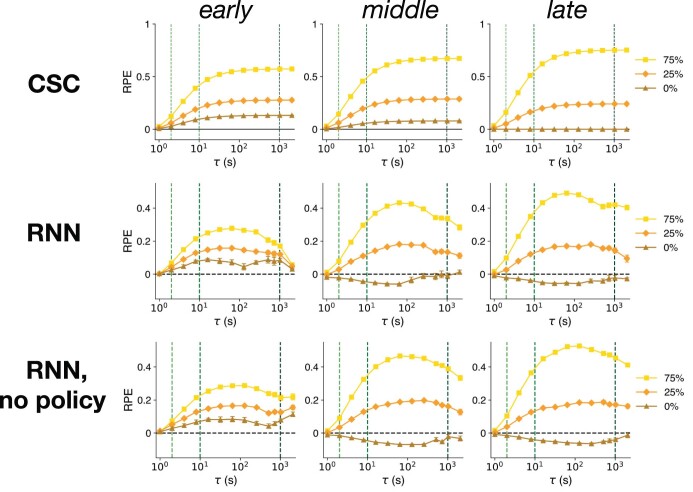
Effects of extended model training on cue discrimination with different discount factors. Top row, cue-evoked RPEs in the CSC model at “early” (600 training steps), “middle” (1000) and “late” (3800) stages of learning, as a function of the time parameter τ. (*γ* = *e*^*−dt/τ*^, where dt is the time step size, here 100 ms). Green dashed lines mark *γ* = 0.95, 0.99 and 0.9999 as used in Extended Data Fig. 5. Note that for low γ, all cue responses are small even after learning since any potential reward is heavily discounted. This CSC model initially shows a positive response to the 0% cue due to overlapping cue representations; over training this response fades to zero (but cannot become negative). Middle row, same for an RNN model (early = 100, middle = 750, late = 1400 training steps, with dt = 50 ms). To isolate the effect of varying time scale τ, this model variant used just a single network (a single τ) rather than three. Note that at early and middle stages of learning, if τ is large (γ is close to 1) the RNN model shows less discrimination between cues compared to intermediate τ (γ), consistent with the observed difference between VS and DMS. Bottom row, same as middle row, but also removing the Actor (poking) component. Data are presented as mean ± SEM.

With extended RNN training, the ‘DLS’ and ‘DMS’ responses to cues remained relatively stable, but ‘VS’ cue discrimination continued to improve, eventually also acquiring negative RPE responses to the 0% cue (Extended Data Fig. 6). In other words, a long time horizon made learning slow, consistent with prior observations in RL models^58^. With hindsight, this made intuitive sense. If the effective time horizon encompasses many trials, it will include multiple rewards regardless of which cue is presented on a given trial (Fig. 4b). Correctly assigning value to particular cues is therefore harder, and the discrimination is slower to learn. By contrast, if the time horizon is comparable to the duration of a single trial (as we suggest for DMS), the average outcomes following distinct cues are very different (closer to the nominal 75%, 25% and 0%) and so learning the distinct associated values can be more quickly accomplished.

The idea of distinct timescales thus provides a concise explanation for the subregional differences in cue-evoked DA transients. DLS responses are weaker because the cues indicate a reward that is too far away in time, given a short time horizon. VS responses are slower to discriminate, because the rewards that follow each cue are not very different, over a long time horizon. DMS shows stronger, well-discriminating responses because its intermediate time horizon best matches the actual timescale of predictions provided by the Pavlovian cues.

### Region-specific discounting in a multiple delay task

**Fig. 5:**
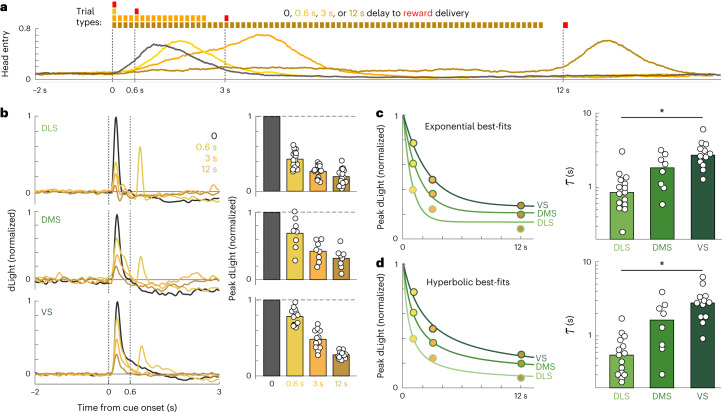
Subregion-specific discount rates in a multiple delay task. **a**, Top, the task has four trial types (chosen at random), each with a distinct delay to reward. Colored bars indicate tone pips. Bottom, average pattern of head entries after training (*n* = 15 sessions, from five rats each recorded on training days 15, 20 and 25). **b**, Left, average dLight signals aligned to the onset of each cue (same sessions as **a**; recording locations *n* = 15 DLS, 8 DMS and 12 VS). Signals are normalized to the peak response to unpredicted reward delivery (that is zero delay) in the same session. All subregions show the same ordering of cue response sizes but differ in their relative sizes. The second peak visible for 0.6 s trials is the response to the reward delivery cue. Right, quantification of peak dLight DA (within 0.5 s of cue onset), with circles indicating averages for individual sessions. This peak depends significantly on both cue identity and subregion (two-way ANOVA, significant CUE × AREA interaction, *F*(4, 96) = 29.3, *P* = 1.3 × 10^−22^). **c**, Left, fit of average responses to different cues, assuming exponential discounting of future rewards. Right, best-fit exponential decay rate *τ* for each session (circles) for each subregion. *τ* depends significantly on subregion (one-way ANOVA, *F*(2, 32) = 13.6, *P = 5.2 × 10^−5^). **d**, Same as **c**, but assuming hyperbolic discounting of future rewards. *τ* depends on subregion (one-way ANOVA, *F*(2, 32) = 12.8, *P = 7.9 × 10^−^^5^).

To confirm that different striatal subregions discount future rewards at different rates, we ran another experiment (in a new cohort of rats). This time, the distinct tone cues indicated distinct delays to potential reward delivery (0.6, 3 and 12 s) rather than different probabilities. After training, rats distinguished between cues in their anticipatory head entries to the food port (Fig. 5a). Furthermore, in all subregions the magnitude of the DA response was greater for cues indicating sooner, rather than later, reward (Fig. 5b), consistent with prior work^34,59,60^. However, the responses were not identical between subregions—for example, in VS the response to the cue indicating a brief delay (0.6 s) was only slightly smaller than to zero delay, while in DLS it was much smaller (Fig. 5b). We used these cue responses to estimate a discount rate, by fitting either exponential (Fig. 5c) or hyperbolic (Fig. 5d) discounting curves^61^. In each case, we found the fastest discounting in DLS and the slowest in VS, consistent with our earlier results.

## Discussion

Here we have demonstrated a consistent ordering of timescales—DLS fastest, DMS intermediate and VS slowest—across three very distinct functional properties of DA transients. This raises the important question of how these properties are related to each other. Why should a more rapid pace of DA fluctuations in DLS accompany faster discounting of future rewards? Why should slower discounting by VS DA accompany more prolonged integration of past rewards?

**Extended Data Fig. 7:**
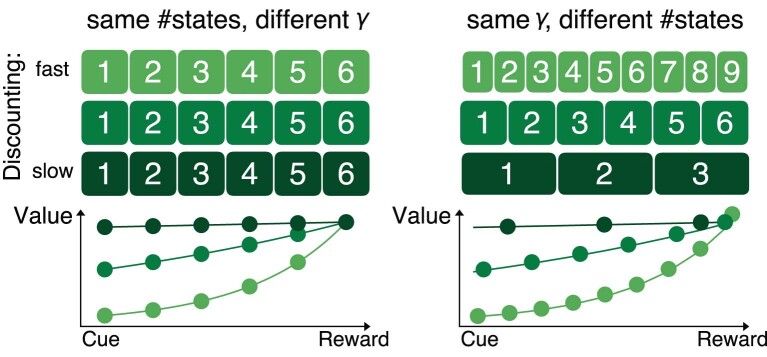
Apparent discount rates can reflect the tempo of state transitions. Discounting differences could be produced by applying a different discount rate γ at each state transition (left), or by applying the same discount rate over a different number of state transitions within a given interval (right). For illustrative purposes, this cartoon assumes a discrete set of defined states in each case.

As noted earlier, one key factor may be the distinct functional representations across hierarchical levels of cortical-basal ganglia circuits^31,32,62,63^. DLS preferentially contributes to briefer, simpler movements that can occur in rapid succession and require immediate feedback^64^. This faster tempo of information processing is supported by various features of DLS microcircuitry, including a higher proportion of fast-spiking interneurons to dictate fine spike timing^65^ and quicker DA reuptake to ensure error signals are very brief. Changes in DLS spiking are also typically brief^66,67^, resulting in a rapidly evolving ‘state’ of DLS networks. Such rapid state changes may naturally produce a more limited time horizon. For example, if a fixed discount factor were applied at each discrete state transition, a greater frequency of transitions would produce a faster effective discount rate (Extended Data Fig. 7).

This perspective on DLS functions is complementary to evidence that DLS is involved in ‘habitual’ stimulus–response (S–R) associations^38,68^. The key feature of S–R habits is that they do not take into consideration the future outcomes produced by actions—but in many behavioral situations, those outcomes may be simply too remote in time to be relevant to DLS calculations.

By contrast, VS neurons typically show more prolonged and/or abstract representations^67,69^. The more slowly changing state of VS is likely needed to help maintain a program of behavior over longer timescales^62^. Less-frequent transitions between states result in fewer opportunities for error signals (hence fewer spontaneous DA events) and less need to ensure error signals are brief to avoid overlap with multiple state transitions. Although some imaging studies have suggested that VS circuits discount especially rapidly^33^, our results are instead consistent with an extensive literature demonstrating a critical role for VS in avoiding impulsive behavior^70^, by promoting work to obtain delayed rewards^71–73^.

Our Pavlovian task used a standard systems neuroscience approach—cues that convey information about individual trials, with many trials in each session. However, our results emphasize that animals, as well as their neural sub-circuits, do not necessarily process information in a corresponding trial-based manner^74^. Slower discounting in VS may be important to motivate prolonged work but can retard learning about cues that only provide information about the next few seconds. A VS time horizon that can span multiple trials may also explain puzzling observations of a large VS DA transient as each session begins^75^. If the onset of the first trial indicates that the animal is likely to receive multiple rewards ‘soon,’ from the VS perspective, this should generate a correspondingly large RPE.

A longer time horizon for future rewards in VS was matched by a longer horizon for tracking past rewards. A relationship between past and future reward estimation has been previously proposed by some theories of decision-making and time perception^3^. However, this relationship is not obvious within standard RL theory, for which the discount rate (*γ*) for future rewards is independent of the learning rate (*α*) that determines the timescale over which past rewards affect current reward expectations. One possibility is that the past horizon scales with the future horizon simply due to the need for adequate data sampling. For example, predicting the rewards to come over the next minute is likely to be more accurate given multiple samples of recent 1-min epochs. Obtaining sufficient data may explain why, for each subregion, the estimated past horizon can be longer than the estimated future horizon. Furthermore, estimating further into the future requires tracking rewards proportionately further back into the past, to achieve an equivalent number of past samples.

We used the fact that phasic DA responses to cue onsets can encode RPE to probe underlying reward expectations. However, there are other aspects of DA release dynamics that appear separate to RPE coding and are thus not accounted for by the RPE-focused models we used here. In particular, overall VS DA release may be lower during prolonged epochs of lower reward availability^43,76^, even when the spiking of midbrain DA cells is unchanged^11,77^. Conversely, VS DA can ramp up as animals approach rewards^43,78^, directly reflecting the increasing expectation of reward^79^. These relationships to reward expectation appear to be VS-specific^11^, despite our incorporating distinct subregional timescales for reward rate calculation (Fig. 2a). This aspect of VS DA signaling is likely related to ongoing motivation and vigor and may involve local striatal control of DA release^80,81^. Further investigation of the mechanisms and timescales supporting motivation-related DA release across striatal subregions is beyond the scope of the present work but will be the focus of later studies.

Furthermore, while making multiple reward predictions may be necessary to support a broad range of adaptive behaviors^21,82^, we do not address how the brain may arbitrate between them^83^. Cortical-basal ganglia circuits are not strictly segregated but rather show convergence and connection^30^ consistent with overlapping contributions to behavioral control. A multiplicity of discount rates has been previously proposed^19^ to be responsible for choices that are inconsistent over time, a well-established feature of animal and human economic behavior^84,85^. An important question for future research is whether our increasing impatience as rewards draw near reflects the progressive engagement of more myopic DA-dependent valuation systems.

## Methods

### Animals and behavior

All animal procedures were approved by the University of California, San Francisco Animal Care Committee (protocol AN196232). Twenty adult wild-type Long-Evans rats (15 males) were bred in-house, maintained on a reverse 12-h light/12-h dark cycle and tested during the dark phase. All recordings were performed in an operant chamber (Med Associates) controlled using custom software in LabVIEW 2017. Details on instrumental and Pavlovian behavioral tasks have been published previously^11,43^. For the Pavlovian task, each cue tone (2, 5 or 9 kHz) was presented as a train of pips (100 ms on and 50 ms off) for a total duration of 2.6 s followed by a delay period of 500 ms. Trials with one of the three cues, or an unpredicted reward delivery (sugar pellet, with an audible food hopper click), were delivered in pseudorandom order with a variable intertrial interval (15–30 s, uniform distribution). Instrumental task sessions used the following parameters: left–right reward probabilities were (independently varying, randomly selected) 10%, 50% or 90% for blocks of 35–45 trials; the hold period before the Go cue was 500–1,500 ms (uniform distribution). For included recording sessions, the mean number of trials was 300 (range: 164–407).

For the multiple delay task, we again used cues 2, 5 or 9 kHz tone pips (100 ms duration, 50 ms between pips), with each pitch corresponding to a different delay period (selected at random for each rat). The shortest delay was signaled by a single pip, the intermediate delay by 17 pips and the longest delay comprised 76 pips (totaling 11.4 s). Each pip train was followed by a fixed 0.5 s trace period and then the same sugar pellet reward delivery (at 75% reward probability for all three cues). Sixty trials of each type were randomly intermixed with unpredictable reward delivery. Intertrial intervals were randomly chosen from a uniform distribution between 15 and 30 s.

### Virus and photometry

Under isoflurane anesthesia, 1 μl of adeno-associated virus AAV-DJ-CAG-dLight1.3b (2 × 10^12^ viral genomes per ml; Vigene Biosciences) was slowly (100 nl min^−1^) injected (Nanoject III; Drummond) through a glass micropipette targeting multiple striatal subregions—ventral (anterior–posterior, AP: 1.7, medial–lateral, ML: 1.7, dorsal–ventral, DV: 7.0 mm relative to bregma), dorsomedial (AP: 1.5, ML: 1.8, DV: −4.3) and dorsolateral (AP: 0.84, ML: 3.8, DV: −4.0). During the same surgery, optical fibers (400 μm core and 430 μm total diameter) attached to a metal ferrule (Doric) were inserted (target depth 200 μm higher than AAV) and cemented in place. Data were collected >3 weeks later, to allow for dLight expression. For dLight excitation, blue (470 nm) and violet (405 nm; isosbestic control) light-emitting diodes were alternately switched on and off in 10 ms frames (4 ms on and 6 ms off)^87^. Excitation power at the fiber tip was set to 30 μW for each wavelength. Both excitation and emission signals passed through Mini Cube filters (Doric), and bulk fluorescence was measured with a Femtowatt detector (Newport, Model 2151) sampling at 10 kHz. Time-division multiplexing produced separate 470 nm (DA) and 405 nm (control) signals, which were then rescaled to each other via a least-square fit^88^. For the simultaneous recording of three areas, we used a Neurophotometrics system^89^; technical details were very similar except that the control wavelength was 415 nm and detection was camera-based, sampling at 100 Hz. The fractional fluorescence signal (*dF*/*F*) was then defined as (470–control_fit)/control_fit.

DA fluctuations alter dLight fluorescence, but absolute fluorescence levels are also influenced by several factors that cannot be readily accounted for (such as the extent of viral expression and the precise placement of the fiber). Consequently, raw photometry signals are not directly comparable between subjects (or areas within subjects). We therefore chose to normalize evoked dLight responses within each subject and subregion before calculating averages. In the case of Pavlovian and multiple delay tasks, the dLight signal was normalized to the mean peak response (within 1 s) to unpredictable reward delivery (that is, zero delay trials). For the instrumental task, normalization was done using the peak DA magnitude (within 1 s) following reward delivery (at the Side-In nose poke). The DA response to cues was then estimated as the maximum or the minimum normalized response within 0.5 s after cue onset, whichever had the larger absolute value (using a 1 s window instead did not change results).

### Histological confirmation

To verify probe placements, animals were perfused transcardially with PBS and then 4% PFA. Brains were postfixed in 4% PFA for 24 h, then placed in 30% sucrose in PBS for >48 h and sectioned at a 100 μm thickness with a microtome. We used immunofluorescence staining to visualize dLight expression. Brain sections with probe placement were identified and then blocked in a 0.4% Triton X-100 solution with 5% normal goat serum for 1 h at room temperature, followed by overnight incubation in a rabbit anti-green fluorescent protein (GFP) primary antibody solution (Abcam, ab290; 1:1,000) in PBS in a cold room. Sections were washed three times in PBS for 10 min at room temperature and incubated in an Alexa 488-conjugated goat anti-rabbit secondary antibody solution (1:250) in PBS for 1 h at room temperature. Finally, sections were washed six times in PBS for 5 min at room temperature and then mounted onto glass slides and coverslipped using Fluoromount-GTM Mounting Medium, with DAPI. Fluorescent images were taken using a fluorescence microscope (Keyence BZ-X810) with a ×2 objective lens. Fiber tip locations from both hemispheres were projected onto the same side in the atlas space.

### Computational models

#### Trial-level models

For the time-based leaky integrator, the reward rate was incremented by 1 at each time the rat received a reward and exponentially decayed with time constant *τ* using ${{{{\rm{d}}}V}}_{t}/{{{{\rm{d}}}t}}=-\tau +r(t)$, where *r*(*t*) equals one when a trial is rewarded and zero otherwise. *τ* was varied between 1 and 2,500 s, to find the strongest negative correlation between reward rate and the DA peak after Side-In (within 0–1 s, on rewarded trials; that is positive RPE coding). To estimate the learning rate, we instead used a trial-based delta rule. This model tracks a value that is updated once per trial by *V*(*t*) = *V*(*t* − 1) + *α* (*r* − *V*(*t* − 1)), where *V*(*t*) is the trial value at trial *t*, *α* is the learning rate and *r* is the outcome of each trial (0 or 1). To find the best fit, we varied *α* between 0 and 1 (in 0.01 steps).

To estimate the discounting time constant (*τ*) in the multiple delay task, we fit either an exponential ($f=b+{{Ae}}^{-t/\tau }$) or a hyperbolic ($f=b+A/(1+t/\tau$)) curve to the peak DA response evoked by each cue. For simplicity, in Fig. 5 we ignore the 75% probability of reward. However, the ordering of subregions was preserved if we adjusted for probability by scaling the cue responses or if we omitted the baseline term *b*.

#### Real-time models

The CSC model is a standard temporal-difference model of conditioning^49^. Values are defined as a linear function of features **x** and weights $w$, ${{{V}}}_{{{t}}}\left({\mathbf{x}}\right)={{{w}}}_{{{t}}}{\mathbf{x}}={\sum }_{{{i}}=1}^{{{n}}}{{{w}}}_{{{t}}}({{i}}){\mathbf{x}}({{i}})$, where *n* is the time steps in a trial. The vector **x** is nonzero only at the *t*-th element at time step *t* after cue onset, that is, ${{\mathbf{x}}}\left(i\right)={\delta }_{{{it}}}$, where ${\delta }_{{{it}}}$ is the Kronecker δ function. In addition to activating a single distinct feature for each cue, we also included one shared feature activated by any of the three cues, to allow for generalization. The weights $w$ update according to ${w}_{t+1}={w}_{t}+\alpha {\delta }_{t}{e}_{t}$, where *α* is the learning rate (we used *α* = 0.01), ${\delta }_{t}$ is the RPE and *e*_*t*_ is an eligibility trace. The RPE is defined as ${\delta }_{t}={r}_{t}+\gamma {V}_{t}\left({\mathbf{x}}_{t}\right)-{V}_{t}({\mathbf{x}}_{t-1})$, where *γ* is the discount factor. The eligibility trace *e*_*t*_ is included to accelerate learning and updated by ${e}_{t+1}=\gamma \lambda {e}_{t}+{x}_{t}$, where *λ* is a decay factor (we used *λ* = 0.98). The CSC model was run separately for each discount factor.

The RNN model, based on an advantage actor-critic architecture^90^, is composed of LSTM (long short-term memory) units^91^. These are organized as three subnetworks (‘DLS’, ‘DMS’ and ‘VS’) of 32 nodes each, with internal recurrent connections but without direct connections between subnetworks. Each subnetwork receives the same copy of the sensory inputs at each time point and generates its own value estimate using a distinct discount factor. All three subnetworks project to the same policy component, together generating the probability for taking an action (either ‘poke’ or ‘no-poke’). These probabilities are sampled to determine the action at each time step. We used a time step of 50 ms.

The vector of sensory inputs to the RNN includes the food delivery click (0 for no-click or 5 for click), auditory cues and background dimensions. Background dimensions (*n* = 3, all set constantly to 1) are included to mimic the background or contextual inputs to the network. The auditory cues consist of 20 dimensions, of which three are the distinctive one-hot features of the three cues and the remainder are set to 1 during all cue presentations to produce similarity between cues.

At each time step, the RNN model receives reward feedback. Before reward delivery, the reward is 0 for taking the action ‘no-poke’ and −0.003 for taking the action ‘poke’; that is, there is a small poking cost to discourage constant poking. If the poke output is maintained on consecutive time steps, the cost is reduced to 10% of that for the first poke. In a rewarded trial, the reward (with value 1) is collected by the first ‘poke’ action after the reward delivery click. We adopted the convention^4^ that the reward associated with an action ${a}_{t}$ at time *t* is denoted as ${r}_{t+1}$.

The network was trained to perform the conditioning task by minimizing a loss function with three terms,

$${L}_{{\mathrm{PPO}}}^{\theta }={{\mathbb{E}}}_{t}[{L}_{t}^{P}\left(\theta \right)+{\beta }_{V}{L}_{t}^{V}\left(\theta \right)-{\beta }_{e}{L}_{t}^{e}\left(\theta \right)],$$

where the expectation was over a sequence of time steps with length *T*. We used *T* = 10,000 steps, which encompasses multiple (~20) trials. We took the proximal policy optimization (PPO) for estimating the policy loss, which has the following form^92^:

$${{{L}}}_{{{t}}}^{{{P}}}\left({{\theta }}\right)=\min ({{{\rho }}}_{{{t}}}{{{A}}}_{{\rm{t}}},{\rm{clip}}\left({{{\rho }}}_{{{t}}},1-{{\epsilon }},1+{{\epsilon }}\right){{{A}}}_{{{t}}}),$$

where ${{{\rho }}}_{{{t}}}=\frac{{{{\pi }}}_{{{\theta }}}({{{a}}}_{{{t}}}{\rm{|}}{{{s}}}_{{{t}}})}{{{{\pi }}}_{{{{\theta }}}_{{\rm{old}}}}({{{a}}}_{{{t}}}{\rm{|}}{{{s}}}_{{{t}}})}$ is the probability ratio, whose value is clipped with a parameter ${{\epsilon }}$. The advantage *A*_*t*_ includes three components,

$${{{A}}}_{{{t}}}={{{A}}}_{{\rm{VS}}}^{{\rm{GAE}}}\left({{t}}\right)+{{{A}}}_{{\rm{DMS}}}^{{\rm{GAE}}}\left({{t}}\right)+{{{A}}}_{{\rm{DLS}}}^{{\rm{GAE}}}\left({{t}}\right),$$

where each term is the generalized advantage estimator (GAE)^93^ from one of the three subnetworks. Take the VS term as an example and define ${{{\delta }}}_{{{t}}}^{{\rm{VS}}}={{{r}}}_{{{t}}+1}+{{{\gamma }}}_{{\rm{VS}}}{{{V}}}_{{{t}}+1}^{{\rm{VS}}}-{{{V}}}_{{{t}}}^{{\rm{VS}}}$ as the RPE at time *t*, then

$${{{A}}}_{{\rm{VS}}}^{{\rm{GAE}}}\left({{t}}\right)={{{\delta }}}_{{{t}}}+\left({{{\gamma }}}_{{\rm{VS}}}{{\lambda }}\right){{{\delta }}}_{{{t}}+1}+\cdots +{\left({{{\gamma }}}_{{\rm{VS}}}{{\lambda }}\right)}^{{{T}}-{{t}}}{{{\delta }}}_{{{T}}},$$

where *T* is the sequence length and *λ* is a GAE parameter, analogous to the *λ* in the TD(*λ*) algorithm^93^. The RPE to be compared with the DA signals is defined as ${{\rm{RPE}}}^{{\rm{VS}}}\left({{t}}\right)={{{r}}}_{{{t}}}+{{{\gamma }}}_{{\rm{VS}}}{{{V}}}_{{{t}}}^{{\rm{VS}}}-{{{V}}}_{{{t}}-1}^{{\rm{VS}}}$.

The value loss was given by

$${{{L}}}_{{{t}}}^{{{V}}}({{\theta }})={\left({\bar{{{r}}}}_{{{t}}}^{{\rm{VS}}}-{{{V}}}_{{{t}}}^{{\rm{VS}}}({{\theta }})\right)}^{2}+{\left({\bar{{{r}}}}_{{{t}}}^{{\rm{DMS}}}-{{{V}}}_{{{t}}}^{{\rm{DMS}}}({{\theta }})\right)}^{2}+{\left({\bar{{{r}}}}_{{{t}}}^{{\rm{DLS}}}-{{{V}}}_{{{t}}}^{{\rm{DLS}}}({{\theta }})\right)}^{2},$$

where ${\bar{{{r}}}}_{{{t}}}^{{\rm{VS}}}$, ${\bar{{{r}}}}_{{{t}}}^{{\rm{DMS}}}$ and ${\bar{{{r}}}}_{{{t}}}^{{\rm{DLS}}}$ are the accumulated discounted rewards within the sequence, given the corresponding discount factor for each subnetwork. We used the value right after *T* to bootstrap the contribution from rewards beyond this sequence. For instance, the expected reward for VS has the following expression:

$${\bar{{{r}}}}_{{{t}}}^{{\rm{VS}}}={{{r}}}_{{{t}}+1}+{{{\gamma }}}_{{\rm{VS}}}{{{r}}}_{{{t}}+2}+\cdots +{{{\gamma }}}_{{\rm{VS}}}^{{{T}}-1}{{{r}}}_{{{t}}+{{T}}}+{{{\gamma }}}_{{\rm{VS}}}^{{{T}}}{{{V}}}_{{{t}}+{{T}}}.$$

Because ${{{\gamma }}}_{{\rm{VS}}}$ is very close to 1, the accumulated reward for ‘VS’ subnetwork reflects contributions from multiple trials. Faster discounting for ‘DMS’ and (especially) ‘DLS’ subnetworks results in minimal contributions from subsequent trials. The entropy term *L*^*e*^ represents the entropy of the probability distribution of taking the two actions and was added to encourage exploration^90^. The parameters used were as follows: ${{{\beta }}}_{{{V}}}=0.8$, ${{{\beta }}}_{{{e}}}=0.001$ and ${{\lambda }}=0.98$. The discount factor $\gamma$ and time constant $\tau$ are related by $\gamma ={e}^{-{{{\rm{d}}t}}/\tau }$, where d*t* is the time step and $\tau$ for the three areas ('DLS', 'DMS', 'VS') was set to 2 s, 10 s and 1,000 s, respectively. The weights of the network were updated using the Adam method^94^, with a learning rate of 0.0005.

### Statistics and reproducibility

No statistical methods were used to predetermine sample sizes, but our sample sizes are similar to those reported in previous publications^11,28,76^.

We removed from analyses six fiber placements that produced consistently weak DA signals (3 DMS and 3 VS), and we also excluded all other individual sessions for which the mean peak DA response to unexpected reward cues was less than 1 s.d. (*z* < 1; 20 of 435 fiber-sessions excluded, 2 DLS, 16 DMS, 2 VS). Data collection and analysis were not performed blind to the conditions of the experiments. Stimulus presentations and trial outcomes were randomized by computer. Data distribution was assumed to be normal, but this was not formally tested.

### Reporting summary

Further information on research design is available in the Nature Portfolio Reporting Summary linked to this article.

## Online content

Any methods, additional references, Nature Portfolio reporting summaries, source data, extended data, supplementary information, acknowledgements, peer review information; details of author contributions and competing interests; and statements of data and code availability are available at 10.1038/s41593-023-01566-3.

## Supplementary information

Reporting Summary

Supplementary Data Editorial assessment report.

Supplementary Video 1 Example of simultaneously recorded DA signals across striatal subregions, illustrating distinct tempo of fluctuations.

## Data Availability

The data have been made publicly available at 10.5061/dryad.00000008m.

## Code availability

The custom code used for simulation and data analysis is available on the Berke Lab GitHub page: https://github.com/Berke-lab.
