# Supplementary material for: Dopamine transients follow a striatal gradient of reward time horizons
Source: Nat Neurosci. 2024 Feb 6;27(4):737–46. doi: 10.1038/s41593-023-01566-3 (PMC11001583; doi:10.1038/s41593-023-01566-3)
Supplement: Supplementary file 1 — Reporting Summary [file 41593_2023_1566_MOESM1_ESM.pdf]

nature portfolio

Corresponding author(s): BERKE

Last updated by author(s): Dec 14, 2023

Reporting Summary

Nature Portfolio wishes to improve the reproducibility of the work that we publish. This form provides structure for consistency and transparency in reporting. For further information on Nature Portfolio policies, see our Editorial Policies and the Editorial Policy Checklist.

Statistics

For all statistical analyses, confirm that the following items are present in the figure legend, table legend, main text, or Methods section.

n/a	Confirmed	
☐	☒	The exact sample size (*n*) for each experimental group/condition, given as a discrete number and unit of measurement
☐	☒	A statement on whether measurements were taken from distinct samples or whether the same sample was measured repeatedly
☐	☒	The statistical test(s) used AND whether they are one- or two-sided<br>*Only common tests should be described solely by name; describe more complex techniques in the Methods section.*
☐	☒	A description of all covariates tested
☐	☒	A description of any assumptions or corrections, such as tests of normality and adjustment for multiple comparisons
☐	☒	A full description of the statistical parameters including central tendency (e.g. means) or other basic estimates (e.g. regression coefficient) AND variation (e.g. standard deviation) or associated estimates of uncertainty (e.g. confidence intervals)
☐	☒	For null hypothesis testing, the test statistic (e.g. *F*, *t*, *r*) with confidence intervals, effect sizes, degrees of freedom and *P* value noted<br>*Give P values as exact values whenever suitable.*
☒	☐	For Bayesian analysis, information on the choice of priors and Markov chain Monte Carlo settings
☒	☐	For hierarchical and complex designs, identification of the appropriate level for tests and full reporting of outcomes
☒	☐	Estimates of effect sizes (e.g. Cohen's *d*, Pearson's *r*), indicating how they were calculated

Our web collection on statistics for biologists contains articles on many of the points above.

Software and code

Policy information about availability of computer code

Data collection	Custom LabView 2017 code for behavioral control and data acquisition; Neurophotometrics built-in software for subset of data acquisition
Data analysis	Custom code: Matlab R2021 for data processing. Python 3.8 for data analysis. Tensorflow 1.1X for network model.

For manuscripts utilizing custom algorithms or software that are central to the research but not yet described in published literature, software must be made available to editors and reviewers. We strongly encourage code deposition in a community repository (e.g. GitHub). See the Nature Portfolio guidelines for submitting code & software for further information.

Data

Policy information about availability of data

All manuscripts must include a data availability statement. This statement should provide the following information, where applicable:

- Accession codes, unique identifiers, or web links for publicly available datasets
- A description of any restrictions on data availability
- For clinical datasets or third party data, please ensure that the statement adheres to our policy

Data availability: The data has been made publicly available on https://doi.org/10.5061/dryad.00000008m.
Code availability: The custom code used for simulation and data analysis are available on the Berke lab Github page: https://github.com/Berke-lab

Research involving human participants, their data, or biological material

Policy information about studies with human participants or human data. See also policy information about sex, gender (identity/presentation), and sexual orientation and race, ethnicity and racism.

Reporting on sex and gender	N/A
Reporting on race, ethnicity, or other socially relevant groupings	N/A
Population characteristics	N/A
Recruitment	N/A
Ethics oversight	N/A

Note that full information on the approval of the study protocol must also be provided in the manuscript.

Field-specific reporting

Please select the one below that is the best fit for your research. If you are not sure, read the appropriate sections before making your selection.

☒ Life sciences ☐ Behavioural & social sciences ☐ Ecological, evolutionary & environmental sciences

For a reference copy of the document with all sections, see nature.com/documents/nr-reporting-summary-flat.pdf

Life sciences study design

All studies must disclose on these points even when the disclosure is negative.

Sample size	No statistical methods were used to pre-determine sample sizes but our sample sizes are similar to those reported in previous publications (11,28,76)
Data exclusions	We removed from analyses 6 fiber placements that produced consistently weak DA signals (3 DMS, 3 VS), and we also excluded all other individual sessions for which the mean peak DA response to unexpected reward cues was less than one standard deviation ($Z < 1$; 20 of 435 fiber-sessions excluded, 2 DLS, 16 DMS, 2 VS).
Replication	No replications were attempted.
Randomization	Animals were not assigned to different groups.
Blinding	Investigators were not blinded. The specific random sequences of stimuli presented to each rat were controlled by computer, rather than investigators.

Reporting for specific materials, systems and methods

We require information from authors about some types of materials, experimental systems and methods used in many studies. Here, indicate whether each material, system or method listed is relevant to your study. If you are not sure if a list item applies to your research, read the appropriate section before selecting a response.

Materials & experimental systems

n/a	Involved in the study	
☐	☒	Antibodies
☒	☐	Eukaryotic cell lines
☒	☐	Palaeontology and archaeology
☐	☒	Animals and other organisms
☒	☐	Clinical data
☒	☐	Dual use research of concern
☒	☐	Plants

Methods

n/a	Involved in the study	
☒	☐	ChIP-seq
☒	☐	Flow cytometry
☒	☐	MRI-based neuroimaging

Antibodies

Antibodies used	Rabbit anti-GFP antibody was obtained from Abcam (cat# ab290).<br>Goat Anti-Rabbit Alexa Fluor 488, Abcam (ab150077)
Validation	Information on validation is available at the manufacturer's website: https://www.abcam.com/gfp-antibody-ab290.html

Animals and other research organisms

Policy information about studies involving animals; ARRIVE guidelines recommended for reporting animal research, and Sex and Gender in Research

Laboratory animals	Wild-type Long-Evans rats were bred in-house. Ages ranged from 6-12 months.
Wild animals	N/A
Reporting on sex	Both sexes were used, but the study was not designed to provide adequate statistical power for analysis by sex.
Field-collected samples	N/A
Ethics oversight	All animal procedures were approved by the University of California, San Francisco Animal Care Committee (protocol# AN196232).

Note that full information on the approval of the study protocol must also be provided in the manuscript.

Plants

Seed stocks	N/A
Novel plant genotypes	N/A
Authentication	N/A
