# Supplementary material for: Dopamine transients follow a striatal gradient of reward time horizons
Source: Nat Neurosci. 2024 Feb 6;27(4):737–46. doi: 10.1038/s41593-023-01566-3 (PMC11001583; doi:10.1038/s41593-023-01566-3)
Supplement: Supplementary file 2 — Editorial assessment report. [file 41593_2023_1566_MOESM2_ESM.pdf]

Manuscript details

Tracking number	Submission date	Decision date	Peer review type
GUIDEDOA-22-00466	May 12, 2022	Jul 27, 2022	Single-blind

Manuscript title	Author details
A Spectrum of Time Horizons for Dopamine Signals<br><br>**Preprint:** link if available	Joshua Berke<br><br>Affiliation: **University of California, San Francisco**

Editorial assessment team

**Primary editor**	**Luis Mejia**<br>Home journal: ***Nature Neuroscience***<br>ORCID: **0000-0001-5439-6803**<br>Email: **luis.mejia@us.nature.com**
**Other editors consulted**	**David Rowland**<br>Home journal: ***Nature***<br>ORCID: **0000-0002-2735-2730**<br><br>**Fiona Carr**<br>Home journal: ***Nature Communications***<br>ORCID**: 0000-0002-2957-1371**
**About your primary editor**	Luis joined Nature Neuroscience in 2018. He received his Ph.D. in Neuroscience from Harvard Medical School, followed by postdoctoral research at Cold Spring Harbor Laboratory in the lab of Bo Li, where he studied orbitofrontal-striatal projection neurons in value- and valence-based decisions and behaviors in mice, using in vivo optogenetics

	and calcium imaging. His research interests include systems and circuits neuroscience, and in vivo imaging and neuroscience methods. Luis is based in the New York office.

Editorial assessment and review synthesis

**Editor's summary and assessment**	The authors report that dopamine signals in striatum convey reward prediction errors over a gradient of distinct timescales, accelerating from ventral striatum (slowest) to dorsomedial striatum to dorsolateral striatum (fastest). The authors propose that these timescale differences are related to differences in delay discounting rates. The editors found the work to be of interest and potentially exciting. The editors also had some questions about the scope of data support provided for the novel conclusions.<br><br>As part of the Guided Open Access pilot, editors from Nature, Nature Neuroscience and Nature Communications have discussed the reviewer reports and the manuscript's suitability for the journals. After careful evaluation, our editorial recommendation is to revise the manuscript and submit back through the Guided Open Access submission portal for consideration at Nature Neuroscience or Nature Communications.
**Editorial synthesis of reviewer reports**	Your manuscript has been seen by 3 reviewers with expertise in striatal and dopaminergic signaling and reward and reinforcement learning. While the reviewers find the work to be of interest, they have raised a number of substantial conceptual, technical and analytical critiques.<br><br>To be considered further at *Nature Neuroscience*, you would need to fully address all the reviewer concerns and comments, including those technical points calling for further data and analyses in support of the conclusions.<br><br>To be considered further at *Nature Communications*, you would need to address all the reviewer concerns and comments; the editors would be open to seeing a revised version of the manuscript and would strongly encourage extension of the findings.

Editorial recommendation

Nature<br><br>Revision not invited	Following editorial assessment of the manuscript and reviewer reports, it was felt the advance is not sufficient for further consideration at Nature.
Journal 2<br><br>Major revisions with extension of the work	The editors would expect that all the referees' points be addressed with additional data and analyses.
Journal 3<br><br>Major revisions with extension of the work	The editors would strongly encourage that all the referees' points be addressed with additional data and analyses.

Next steps

**Editorial recommendation 1:**	Our top recommendation is to revise and resubmit your manuscript to *Nature Neuroscience*. We feel that the additional data requested are reasonable.
**Editorial recommendation 2:**	You may also choose to revise and resubmit your manuscript to *Nature Communications*. This option might be best if certain requested data and revisions are not feasible at this time.
**Note**	As stated on the previous page, *Nature* is not inviting a revision at this time. Please keep in mind that the journal will not be able to consider any appeals of their decision through Guided Open Access.

Revision

To follow our recommendation, please upload the revised manuscript files using **the link provided in the decision letter**.

Revision checklist

- ☐ Cover letter, stating to which journal you are submitting
- ☐ Revised manuscript
- ☐ Point-by-point response to reviews
- ☐ Updated Reporting Summary and Editorial Policy Checklist
- ☐ Supplementary materials (if applicable)

Submission elsewhere

If you choose not to follow our recommendations, you can still take the reviewer reports with you.

Option 1: Transfer to another Nature Portfolio journal
Springer Nature provides authors with the ability to transfer a manuscript within the Nature Portfolio, without the author having to upload the manuscript data again. To use this service, **please follow the transfer link provided in the decision letter.** If no link was provided, please contact guidedOA@nature.com.

Note that any decision to opt in to In Review at the original journal is not sent to the receiving journal on transfer. You can opt in to In Review at receiving journals that support this service by choosing to modify your manuscript on transfer.

Option 2: Portable Peer Review option for submission to a journal outside of Nature Portfolio
If you choose to submit your revised manuscript to a journal at another publisher, we can share the reviews with another journal outside of the Nature Portfolio if requested. You will need to request that the receiving journal office contacts us at guidedOA@nature.com. We have included editorial guidance below in the reviewer reports and open research evaluation to aid in revising the manuscript for publication elsewhere.

Annotated reviewer reports

The editors have included some additional comments on specific points raised by the reviewers below, to clarify requirements for publication in the recommended journal(s). However, please note that all points should be addressed in a revision, even if an editor has not specifically commented on them.

Reviewer #1 information	
**Expertise**	Striatum and dopamine neurons, recording and imaging, behavior, RL
**Editor's comments**	The reviewer finds the topic to be of interest, but has raised important conceptual comments in support of the conclusions and a number of important points regarding the technical aspects.

Reviewer #1 comments	
**Section**	**Annotated Reviewer Comments**
**Remarks to the Author: Overall significance**	This study by Mohebi et al involves photometric recordings of dopamine (DA) from the dorso-lateral (DLS), dorso-medial (DMS) and ventral striatum (VS) of rats using a genetically encoded DA sensor. The authors show that recordings in the three regions have varying time constants, and speculate that this may be due to a spectrum in the degree to which future rewards are discounted as a function of time used to learn the value functions from which RPEs are derived. They combine analysis of DA recordings with computational modeling to support their claims. While I find the data to be suggestive as opposed to convincing, and the novelty is somewhat undercut by Kobayashi and Schultz, 2008, and Enomoto et al, 2020, the subject is interesting and of wide interest.<br>**Nature Neuroscience and Nature Communications would ask that you appropriately address all the points.**
**Remarks to the Author: Impact**	I believe the influence on thinking in the field is somewhat undercut by the suggestive nature of the results combined with the existence of previous studies.<br>**Nature Neuroscience and Nature Communications would ask that you appropriately address all the points.**
**Remarks to the Author: Strength of the claims**	I do not believe the authors' have done the best experiments to convincingly demonstrate the claims being made. Specifically, if the authors wished to convincingly test for variable discounting, in my opinion they should have..<br><br>1) done additional experiments to measure dopamine signals while rats perform a

task where delays to reward are systematically varied instead of analyzing data collected in less than ideal settings for the questions at hand in what would appear to be a post-hoc manner.

> **Nature Neuroscience would expect that you appropriately resolve all the points and requests with new data. Nature Communications would strongly encourage that you appropriately resolve the points and requests.**

There is relatively little analysis of behavior in the paper that might be illuminating as to the origin of inter-areal differences in DA response.
2) Did the authors look at behavior more continuously?
3) Do the more rapid fluctuations observed in DLS correspond to behavioral events not under experimenter control?
4) If so, can the modeling approaches employed explain the differential response across areas to these events as well?

> **Nature Neuroscience would expect that you appropriately resolve all the points and requests with new data. Nature Communications would strongly encourage that you appropriately resolve the points and requests.**

The modeling approach strikes me as a bit disjointed. The authors begin by seeking to explain the modest cue response of DLS DA using a reinforcement learning (RL) model containing a complete serial compound (CSC) -type state representation to encode time since cues, together with variable discount rates. However, they then proceed to using recurrent neural networks that were trained to represent value functions with different discount rates. This latter approach would also serve to explain the relatively diminished cue response in the shorter time-constant module, so…
5) why use the CSC-based model at all?

> **Nature Neuroscience would expect that you appropriately resolve all the points and requests with new data. Nature Communications would strongly encourage that you appropriately resolve the points and requests.**

The authors focus on discounting, but variable temporal extendness of state representations might also be expected to produce similar results. Indeed, the different regions of the striatum that are the focus of this study process vastly different types of information (which at least partly extends to the DA neurons that innervate these areas), as determined by the distinct cortical inputs they receive.

6) If one were to use recurrent networks that possessed differing time constants in the information they could represent, not just in the temporal discount of the value functions they were used to learn, would this not produce similar results, even with a fixed discount across networks?

7) Can the authors discriminate between these possibilities?

> **Nature Neuroscience would expect that you appropriately resolve all the points and requests with new data. Nature Communications would**

	**strongly encourage that you appropriately resolve the points and requests.**<br><br>The authors suggest that the time constant of responses follows a particular order, DLS<DMS<VS. However, the cue response in VS in the pavlovian task, even to the cue that predicts the largest probability of reward (the authors do not vary magnitude), is not the largest and the VS discriminates less well between the cues. The authors claim that this can be explained by slower learning due to the longer time constant of discounting in these circuits. This suggests that the animals were not trained for long enough to demonstrate the effect that the authors claim to underlie the variable responses.<br><br>8) It would be more convincing if the authors trained the animals for long enough to demonstrate the effect.<br>**Nature Neuroscience would expect that you appropriately resolve all the points and requests with new data. Nature Communications would strongly encourage that you appropriately resolve the points and requests.**<br><br>Minor comments:<br><br>The authors suggest in the discussion that the circuitry of the basal ganglia is characterized by convergence and cross talk between circuits that are often characterized as being parallel, but then model their recurrent networks without interconnections between the networks possessing different time constants. Which is it? If the recurrent networks are stand ins for cortical networks, surely these should be interconnected.<br><br>Previous work from this group has suggested that DA responses do not encode reward prediction errors, while this study seems all in on the RPE hypothesis of phasic DA. People are allowed to change their minds, but the absence of reference to those previously expressed interpretations is conspicuous.<br>**Nature Neuroscience and Nature Communications would ask that you appropriately address all the points.**

Reviewer #2 information

**Expertise**	Striatum and dopamine neurons, recording and imaging, behavior
**Editor's comments**	The reviewer provides an overall positive assessment of the paper, yet has raised a number of technical critiques regarding the strength of data support for the conclusions made.

Reviewer #2 comments

Section	Annotated Reviewer Comments
**Remarks to the Author: Overall significance**	This paper looks at the interesting question of timescales of DA signals across the striatum. The authors make 3 distinct points about the timescale of DA being faster in DLS than DMS than NAc: 1) DA transients tend to be longer in NAc, 2) learning rate tends to be lower in NAc (more integration of past rewards), 3) temporal discount rates are lower in NAc. I have some concerns, however, about how coherently the data relates to their conclusions, in particular the point on temporal discounting. Ultimately, I think this paper has good potential, but falls short of supporting some of its conclusions (more on that below).<br><br>My second major concern is that there is something confusing about the paper, as written. The intro/motivation and discussion is entirely about temporal discount rates varying across striatum (point 3 above). This contrasts with the fact that many of the figures (and the clearest results) are for points 1&2. This makes the paper a bit confusing to read and it feels a bit misleading. There is no clear link between the 3 “types” of timescales, and only temporal discount is motivated in introduction, or covered in discussion.<br>**Nature Neuroscience and Nature Communications would ask that you appropriately address all the points.**
**Remarks to the Author: Impact**	I think it is a cool hypothesis that could influence thinking in the field. I think the work would be impactful if the strength of the claims were greater.
**Remarks to the Author: Strength of the claims**	1. Regarding their 3 conclusions, the 1st point, that DA transients tend to be longer in NAc, looked quite convincing.<br><br>2. Regrading the 2nd point, that the learning rates are lower in NAc, I have a couple comments/concerns: 1) While they show alpha and tau tend to be different for the various regions, showing some more “raw” form of the data to actually see the effect that previous rewards are weighted less is needed to convince the reader of the result. E.g. if they plot a logistic regression to use previous rewarded and unrewarded choices to predict choice, as in Bayer & Glimcher, to they see the alpha indeed looks different across regions? , 2) the methods for this figure (and the entire paper) are far too sparse. It is explained how alpha is selected for the Rescorla-Wagner model, but not tau for the Sugrue model. Referencing another paper isn’t sufficient. Also, are the model parameters selected per animal? Or per session?<br>**Nature Neuroscience would expect that you address all the questions**

and requests with new data. Nature Communications would strongly encourage that you appropriately address the points and requests.

3. Regarding the 3rd point (the main one about temporal discounting), it doesn’t appear that they did the most straightforward experiment to examine temporal discount rates. If they simply had cues that systematically varied w.r.t. the temporal delay to reward, the fall off in the response to those cues would relate directly to the temporal discount rate. I strongly recommend they perform a more straightforward experiment along those lines to link the DA signal across subregions to temporal discount rates, in order to test their central hypothesis. The problem with doing an experiment that is indirectly testing their results, is that there are also other explanations that are equally consistent with their results. e.g. NAc generalizes between cues more than the other regions, or DMS responds more to cues than other regions.

Nature Neuroscience would expect that you appropriately resolve all the points and requests with new data. Nature Communications would strongly encourage that you appropriately resolve the points and requests.

4. Given they didn’t do the most straightforward test of their hypothesis, they make a more circuitous link between their data and temporal discounting. For example, they fit the TD model in Figure 4, and claim the results look most like DLS data in Figure 3. But in reality, all 3 subergions in 3D look very similar, so it’s not clear what the model in Fig. 4 is showing/testing.

Nature Neuroscience would expect that you appropriately resolve all the points and requests with new data. Nature Communications would strongly encourage that you appropriately resolve the points and requests.

5. The link between FIgure 5 and their conclusions on temporal discounting was also a bit indirect. They find that an RNN trained with different temporal discount rates learned to discriminate cues the “best” for units for which the discount rate was intermediate, i.e. when the discount rate was best matched to the trial lengths. Whereas if the discount rate was too long (meant to model NAc), cross-trial effects interfered with cross-trial effects, and if the discount rate was too short (meant to model DLS), the gap between the cue and the outcomes was long enough that there was a lot of temporal discounting evident that diminished the cue response. While this explanation is reasonable, other explanations are also possible (as mentioned above), which is the disadvantage of not including the experiment that provides the direct test of the hypothesis.

Nature Neuroscience would expect that you appropriately resolve all the points and requests with new data. Nature Communications would strongly encourage that you appropriately resolve the points and requests.

	6. Another concern is that the model in Figure 5 shows very prominent wiggles in the shape of the RPE. I'm not clear what's generating that (does the value function have the corresponding weirdness?). It's not a feature of previous publications using this model, as far as I know, nor is it a feature of the biological DA system. Also, the key result in Figure 5, that the low discount rate had a positive response to cue, is extremely hard to see (in fact all the cue responses are very difficult to see).<br>**Nature Neuroscience would expect that you appropriately resolve all the points and requests with new data. Nature Communications would strongly encourage that you appropriately resolve the points and requests.**<br><br>7. In general, it wasn't clear what "normalized" dlight meant. Please briefly describe normalization in figure legends, since conclusions seem very tied to what responses are being compared to what. Is the same normalization used for model and data?<br>**Nature Neuroscience and Nature Communications would ask that you appropriately address all the points.**
**Remarks to the Author: Reproducibility**	The methods were extremely sparse. Please provide enough detail that someone could reproduce the experiments and analysis, ideally also include a link to code used to generate figures and run models.<br>**Nature Neuroscience and Nature Communications would ask that you appropriately address all the points.**

Reviewer #3 information

**Expertise**	Striatum and dopamine, recording and imaging, behavior, RL
**Editor's comments**	The reviewer finds the work of interest but has raised a number of conceptual issues and points regarding the technical aspects.

Reviewer #3 comments

Section	Annotated Reviewer Comments
**Remarks to the Author: Overall significance**	The paper by Wei et al presents experiments in which the authors recorded DA release across vent and dor striatum, concluding that these signals have a gradient of temporal discount factor.

	major<br>The authors have previously published elegant papers showing that DA release prior to outcome in VS fluctuates over longer times reflecting motivation/value. The current paper replicates some of that results (Fig. 2A lowest panel) and interestingly shows that this pre-outcome coding is absent in the dorsal striatum (same figure 2A). The paper however does not attempt to address this region-dependent difference. Can this difference also be accounted for by different temporal discount factor? If so, does the value/motivation interpretation still hold for this and past results from authors? I think this requires extensive analyses and discussion. As it is, it is unclear to me how interpretation of current results and authors' previous interpretations go together.<br>**Nature Neuroscience would expect that you appropriately resolve all the points and requests with new data. Nature Communications would strongly encourage that you appropriately resolve the points and requests.**<br><br>The manuscript seems to suggest that different dopamine neurons access different value functions (with different discount rate) and use that to compute RPE. The other possibility is that DA neurons have access to the same value function and use different discount rate when reporting RPE. These two possibilities make small mathematical difference and I wonder if authors can separate these? If not, then both interpretation holds?<br>**Nature Neuroscience would expect that you appropriately resolve all the points and requests with new data. Nature Communications would strongly encourage that you appropriately resolve the points and requests.**<br><br>minor<br>VS DA signals remaining positive for all cues (Fig. 3D): looking at figure 3C, it seems that this conclusion depends on the chosen window of analysis. Figure C lowest row for p=0 shows small increase followed by a small longer decrease in DA. If the analysis window is moved to better capture the DA dip, the result might get to zero or even below.<br>**Nature Neuroscience would expect that you appropriately resolve all the points and requests with new data. Nature Communications would strongly encourage that you appropriately resolve the points and requests.**
**Remarks to the Author: Impact**	given recent works on projection-specific DA signals, the paper is appropriate for Nature Communications after a revision.
**Remarks to the Author: Reproducibility**	The data quality is high and this will allow reproducibility.

Open research evaluation

General information

Guidelines for Transparency and Openness Promotion (TOP) in Journal Policies and Practices ("TOP Guidelines")

The recommendations and requests in the table below are aimed at bringing your manuscript in line with common community standards as exemplified by the TOP Guidelines. While every publisher and journal will implement these guidelines differently, the recommendations below are all consistent with the policies at Nature Portfolio. In most cases, these will align with TOP Guidelines Level 2.

FAIR Principles

The goal of the recommendations in the table below related to **data or code** availability is to promote the FAIR Guiding Principles for scientific data management and stewardship (*Scientific Data* **3**: 160018, 2016). The FAIR Principles are a set of guidelines for improving 4 important aspects of digital research objects: **F**indability, **A**ccessibility, **I**nteroperability and **R**eusability.

ORCID

ORCID is a non-profit organization that provides researchers with a unique digital identifier. These identifiers can be used by editors, funding agencies, publishers, and institutions to reliably identify individuals in the same way that ISBNs and DOIs identify books and articles. Thus the risk of confusing your identity with another researcher with the same name is eliminated. **The ORCID website** provides researchers with a page where your comprehensive research activity can be stored.

Springer Nature collaborates with the ORCID organization to ensure that your research contributions (as authors and peer reviewers) are correctly attributed to you. Learn more at https://www.springernature.com/gp/researchers/orcid

Data availability
**Data Availability Statement**
Many journals, including all Nature Portfolio journals, require a Data Availability Statement in the manuscript as a condition of publication. The Data Availability Statement should be as detailed as possible and include accession codes or other unique IDs for deposited data, information about where source data can be found, and specify any restrictions to data access that may apply. At a minimum, the statement should indicate that data are available upon request and explain how data access can be granted. If data access is not possible, the reasons for this must be made clear in the Data Availability Statement.<br><br>More information about the Nature Portfolio data availability policy can be found here: https://www.nature.com/nature-portfolio/editorial-policies/reporting-standards#availability-of-data<br><br>Additional information about Data Availability Statements and Springer Nature's data policies are available here: http://www.springernature.com/gp/authors/research-data-policy/data-availability-statements/12330880
Thank you for including a Data Availability statement in your manuscript. You have stated that data are only available upon request. These statements are strongly discouraged by the research community as they do not make it clear which data are available and under what conditions. The data availability statement must make the conditions of access to the data underlying the study transparent to readers. Please explicitly state if and how readers may access the individual datasets reported in the study, including any unique identifiers or URLs and any restrictions to access that apply.<br><br>See here for more information about Data Availability Statements and Springer Nature's data policies: http://www.springernature.com/gp/authors/research-data-policy/data-availability-statements/12330880

**Other data requests**
In line with community standards regarding open research, Springer Nature strongly supports data sharing and believes that all datasets on which the conclusions of the paper rely should be available to readers. We encourage authors to ensure that their datasets are either deposited in publicly available repositories (where available and appropriate) or presented in the main manuscript or additional supporting files whenever possible.<br><br>To learn more about data sharing and recommended data repositories, please see https://www.springernature.com/gp/authors/research-data-policy/repositories/12327124
All source data underlying the graphs and charts presented in the main figures must be made available as Supplementary Data (in Excel or text format) or via a generalist repository (eg, Figshare or Dryad). This is mandatory for publication in a Nature Portfolio journal, but is also best practice for publication in any venue.

**Data citation**
Please cite (within the main reference list) any datasets stored in external repositories that are mentioned within their manuscript. For previously published datasets, we ask that you cite both the related research article(s) and the datasets themselves. For more information on how to cite datasets in submitted manuscripts, please see our data availability statements and data citations policy:<br>https://www.nature.com/documents/nr-data-availability-statements-data-citations.pdf<br><br>Citing and referencing data in publications supports reproducible research, by increasing the transparency and provenance tracking of data generated or analyzed during research. Citing data formally in reference lists also helps facilitate the tracking of data reuse and may help assign credit for individuals' contributions to research. A number of Springer Nature imprints are signatories of the Joint Declaration on Data Citation Principles, which stress the importance of data resources in scientific communication.

Thank you for depositing your dataset in a public repository. In addition to providing the link within the Data Availability statement, we ask that you also cite the dataset in the main reference list.

Citing and referencing data in publications supports reproducible research, by increasing the transparency and provenance tracking of data generated or analysed during research. Citing data formally in reference lists also helps facilitate the tracking of data reuse and may help assign credit for individuals' contributions to research. A number of Springer Nature imprints are signatories of the Joint Declaration on Data Citation Principles, which stress the importance of data resources in scientific communication.

Code availability and citation

To adhere to community standards and promote transparency in research, any custom software or code should be made publicly available, ideally before publication so that referees can test the code and comment on it.

Please include a statement under the heading "Code Availability", indicating whether and how the custom code/software reported in your study can be accessed, including any restrictions to access. This section should also include information on the versions of any software used, if relevant, and any specific variables or parameters used to generate, test, or process the current dataset. Code availability statements should be provided as a separate section after the Data Availability section.

Upon publication, Nature Portfolio journals consider it best practice to release custom computer code in a way that allows readers to repeat the published results. Code should be deposited in a DOI-minting repository such as Zenodo, Gigantum or Code Ocean and cited in the reference list following the guidelines described in our policy pages (see link below). Authors are encouraged to manage subsequent code versions and to use a license approved by the open source initiative. Full details about how the code can be accessed and any restrictions must be described in the Code Availability statement.

See here for more information about Nature Portfolio's code availability policies: https://www.nature.com/nature-portfolio/editorial-policies/reporting-standards#availability-of-computer-code

We also provide a Code and Software submission checklist that you may find useful: https://www.nature.com/documents/nr-software-policy.pdf

Please note: because of advanced features used in this form, you must use Adobe Reader to open the document and complete it.

Thank you for including a Code Availability statement in your manuscript. However, we noted that you have only indicated that custom code are available upon request. To adhere to community standards and promote transparency in research, the Code Availability Statement must indicate whether and how the code or algorithm can be accessed, including any restrictions to access. Public release of custom software may be required for publication in a Nature Portfolio journal.

Upon publication, Nature Portfolio journals consider it best practice to release custom computer code in a way that allows readers to repeat the published results. Code should be deposited in a DOI-minting repository such as Zenodo, Gigantum or Code Ocean and cited in the reference list following the guidelines described in our policy pages (see link below). Authors are encouraged to manage subsequent code versions and to use a license approved by the open source initiative. Full details about how the code can be accessed and any restrictions must be described in the Code Availability statement.

See here for more information about Nature Portfolio's code availability policies: https://www.nature.com/nature-portfolio/editorial-policies/reporting-standards#availability-of-computer-code

We also provide a Code and Software submission checklist that you may find useful: https://www.nature.com/documents/nr-software-policy.pdf

Please note: because of advanced features used in this form, you must use Adobe Reader to open the document and complete it.

Thank you for making your custom code available via Github. Upon publication, Nature Portfolio journals consider it best practice to release custom computer code in a way that allows readers to repeat the published results. Code should be deposited in a DOI-minting repository such as Zenodo, Gigantum or Code Ocean and cited in the reference list following the guidelines described in our policy pages (see link below). Authors are encouraged to manage subsequent code versions and to use a license approved by the open source initiative.

See here for more information about our code availability policies: https://www.nature.com/nature-portfolio/editorial-policies/reporting-standards#availability-of-computer-code

Ethics

We believe that authors, peer reviewers and editors should be required to disclose any competing interests that might influence their decisions and conclusions around a particular piece of content. In the interests of transparency and to help readers form their own judgements of potential bias, Nature Portfolio journals require authors to declare any competing financial and/or non-financial interests in relation to the work described.

Please provide a 'Competing interests' statement using one of the following standard sentences:

1. The authors declare the following competing interests: [specify competing interests]

2. The authors declare no competing interests.

See the Nature Portfolio competing interests policy for further information: https://www.nature.com/nature-research/editorial-policies/competing-interests

The Springer Nature policy can be found here: https://www.springernature.com/gp/policies/editorial-policies

Further details about the Nature Portfolio policy can be found at https://www.nature.com/commsbio/editorial-policies/ethics-and-biosecurity

Reporting & reproducibility
We believe that research publications should adhere to high standards of transparency and robustness in their methods and results. This, in turn, supports the principle of reproducibility, which is a foundation of good research, especially in the natural sciences. All data that support the conclusions drawn must be presented in the manuscript unless they are published elsewhere.<br><br>Nature Portfolio journals do not allow statements of “data not shown”. Please remove these statements or provide the relevant data.
We believe that research publications should adhere to high standards of transparency and robustness in their methods and results. This, in turn, supports the principle of reproducibility, which is a foundation of good research, especially in the natural sciences.<br><br>The Methods section should contain sufficient detail such that the work could be repeated. It is preferable that all key methods be included in the main manuscript, rather than in the Supplementary Information. Please avoid use of “as described previously” or similar, and instead detail the specific methods used, with appropriate attribution.<br><br>Please note that Nature Portfolio journals allow unlimited space for Methods.
We encourage you to share your step-by-step experimental protocols on a protocol sharing platform of their choice. The Nature Portfolio’s Protocol Exchange is a free-to-use and open resource for protocols; protocols deposited in Protocol Exchange are citable and can be linked from the published article. More details can be found at www.nature.com/protocolexchange/about
**Materials availability**
We encourage you to include within the Methods and/or Data Availability Statement details regarding materials availability for the reported components.
**Statistical reporting**

Wherever statistics have been derived (e.g. error bars, box plots, statistical significance) figure legends should provide and define the n number (i.e. the sample size used to derive statistics) as a precise value (not a range), using the wording "n=X biologically independent samples/animals/cells/independent experiments/n= X cells examined over Y independent experiments" etc. as applicable. The figure legends must also indicate the statistical test used. Where appropriate, please indicate in the figure legends whether the statistical tests were one-sided or two-sided and whether adjustments were made for multiple comparisons. For null hypothesis testing, please indicate the test statistic (e.g. F, t, r) with confidence intervals, effect sizes, degrees of freedom and P values noted.<br><br>All error bars need to be defined in the figure legends (e.g. SD, SEM) together with a measure of centre (e.g. mean, median). For example, the legends should state something along the lines of "Data are presented as mean values +/- SEM" as appropriate. All box plots need to be defined in the legends in terms of minima, maxima, centre, bounds of box and whiskers and percentile.<br><br>For examples of expected description of statistics in figure legends, please see the following: https://www.nature.com/articles/s41467-019-11636-5 or https://www.nature.com/articles/s41467-019-11510-4.<br><br>When describing results as "significant" in the main text, please include details about the statistical test used and provide an exact p-value, rather than a signifcance threshold.
Please note that statistics such as error bars significance and p values cannot be derived from n<3 and must be removed in all such cases.
We strongly discourage deriving statistics from technical replicates, unless there is a clear scientific justification for why providing this information is important. Conflating technical and biological variability, e.g., by pooling technically replicates samples across independent experiments is strongly discouraged.<br><br>For examples of expected description of statistics in figure legends, please see the following: https://www.nature.com/articles/s41467-019-11636-5 or https://www.nature.com/articles/s41467-019-11510-4.

To improve reproducibility of your analyses, please provide details regarding your treatment of outliers.
To improve reproducibility of your analyses, please detail the methods used for data fitting and provide a rationale for this approach.
Bar graphs should only be used to present counts or proportions. If you are using bar graphs that present means/averages, it is best practice to include individual data points and/or convert the graph to a boxplot or dot-plot. You may wish to refer to this blog post (https://ecrlife420999811.wordpress.com/2018/07/10/beyond-bar-graphs-free-tools-and-resources-for-creating-more-transparent-figures-for-small-datasets/) about representing data distribution in plots (particularly for small datasets).
